# Nature of collective decision-making by simple yes/no decision units

**DOI:** 10.1038/s41598-017-14626-z

**Published:** 2017-10-31

**Authors:** Eisuke Hasegawa, Nobuaki Mizumoto, Kazuya Kobayashi, Shigeto Dobata, Jin Yoshimura, Saori Watanabe, Yuuka Murakami, Kenji Matsuura

**Affiliations:** 1 Laboratory of Animal Ecology, Department of Ecology and Systematics, Graduate School of Agriculture, Hokkaido University, Sapporo, 060-8589 Japan; 2 Laboratory of Insect Ecology, Graduate School of Agriculture, Kyoto University, Kyoto, 606-8502 Japan; 3 Graduate School of Science and Technology and Department of Mathematical and Systems Engineering, Shizuoka University, 3-5-1 Johoku, Naka-ku, Hamamatsu, 432-8561 Japan; 4 Marine Biosystems Research Center, Chiba University, Uchiura, Kamogawa, Chiba, 299-5502 Japan; 5 Department of Environmental and Forest Biology, State University of New York College of Environmental Science and Forestry, Syracuse, NY 13210 USA; 6 Graduate School of Medicine, Department of Neuropharmacology, Hokkaido University, Sapporo, 060-8638 Japan; 7 Present Address: Hokkaido Forest Research Station, Field Science Education and Research Center, Kyoto University, 553 Tawa, Shibecha-cho, Kawakami-gun, Hokkaido, 088-2339 Japan

## Abstract

The study of collective decision-making spans various fields such as brain and behavioural sciences, economics, management sciences, and artificial intelligence. Despite these interdisciplinary applications, little is known regarding how a group of simple ‘yes/no’ units, such as neurons in the brain, can select the best option among multiple options. One prerequisite for achieving such correct choices by the brain is correct evaluation of relative option quality, which enables a collective decision maker to efficiently choose the best option. Here, we applied a sensory discrimination mechanism using yes/no units with differential thresholds to a model for making a collective choice among multiple options. The performance corresponding to the correct choice was shown to be affected by various parameters. High performance can be achieved by tuning the threshold distribution with the options’ quality distribution. The number of yes/no units allocated to each option and its variability profoundly affects performance. When this variability is large, a quorum decision becomes superior to a majority decision under some conditions. The general features of this collective decision-making by a group of simple yes/no units revealed in this study suggest that this mechanism may be useful in applications across various fields.

## Introduction

Collective decision-making is an important focus of various scientific fields^1–9^ and particularly focuses on the question of how a collective decision maker achieves the optimal choice from among multiple options^5,9–17^. Most previously proposed mechanisms assume “quality-graded responses” of units (i.e., each response unit returns a larger response to a better option than to a worse option). Although neurons (response units) of the brain (a typical collective decision maker) can make only simple on/off responses based on a response threshold, a brain has the ability to choose the highest quality option from multiple candidates. To achieve the “correct choice,” that is, the choice corresponding to the highest quality among candidates, collective decision makers are required to evaluate the relative quality of options from a collection of binary responses and to then select a single option based on the evaluations.

Nearly a century ago, two seminal studies demonstrated that quantitative evaluation in vision is achieved by a collection of yes/no responses of vision cells with various response thresholds^18,19^. Similarly, it was shown that highly efficient choice is achievable without quality-graded responses by social insect workers^20,21^. These works revealed that relative evaluation by a collective decision maker is achieved by accumulating the individual responses to an option when individuals (response units) have diverse individual acceptance thresholds and do not make direct comparisons among options. Simulation models have also shown that the diverse acceptance thresholds among individuals should be important considerations for ant colonies choosing the best option^20,22,23^. The results of these models are also in accordance with real ants, which exhibit speed-accuracy or speed-cohesion trade-offs^22^. Moreover the previous models also have shown that the decision making by ant workers with threshold variance can work well when the number of individuals is increased, which is consistent with the observations from laboratory experiments^22^. In addition, another study showed that colonies of *Myrmica kotokui* ants preferred a better option (a more condensed sucrose solution) by using only the above-mentioned mechanism^21^. However, as previous studies have mainly focused on the nest emigration system of ants, the fundamental role of the diverse thresholds has not been fully explored. In this study, we investigate the effects of threshold variance on the optimality of decision making using much simplified yes/no response units. Our results are not restricted to nest-site selection in ants, but they can be applicable to broader areas of collective decisions using yes/no units, e.g., neural networks and brains.

We conducted several simulations to elucidate the general features of collective decision-making by yes/no judgement units. We investigated the effects of various factors on the proportion of the correct choice by this collective decision-making mechanism, e.g., the degree of threshold variance, changes in the mean of threshold distributions, choice difficulty, number of units allocated to each option and its variance, effectiveness of a quorum decision, violation of independence of irrelevant alternatives, and conditions for achieving the correct choice. Based on these results, we discuss how a collective decision maker can achieve the correct choice from among multiple options. We also discuss the broad applicability of our mechanism to various fields such as cognitive science, behavioural science, marketing, operations research and consensus making in human societies.

## Results

**Figure 1:**
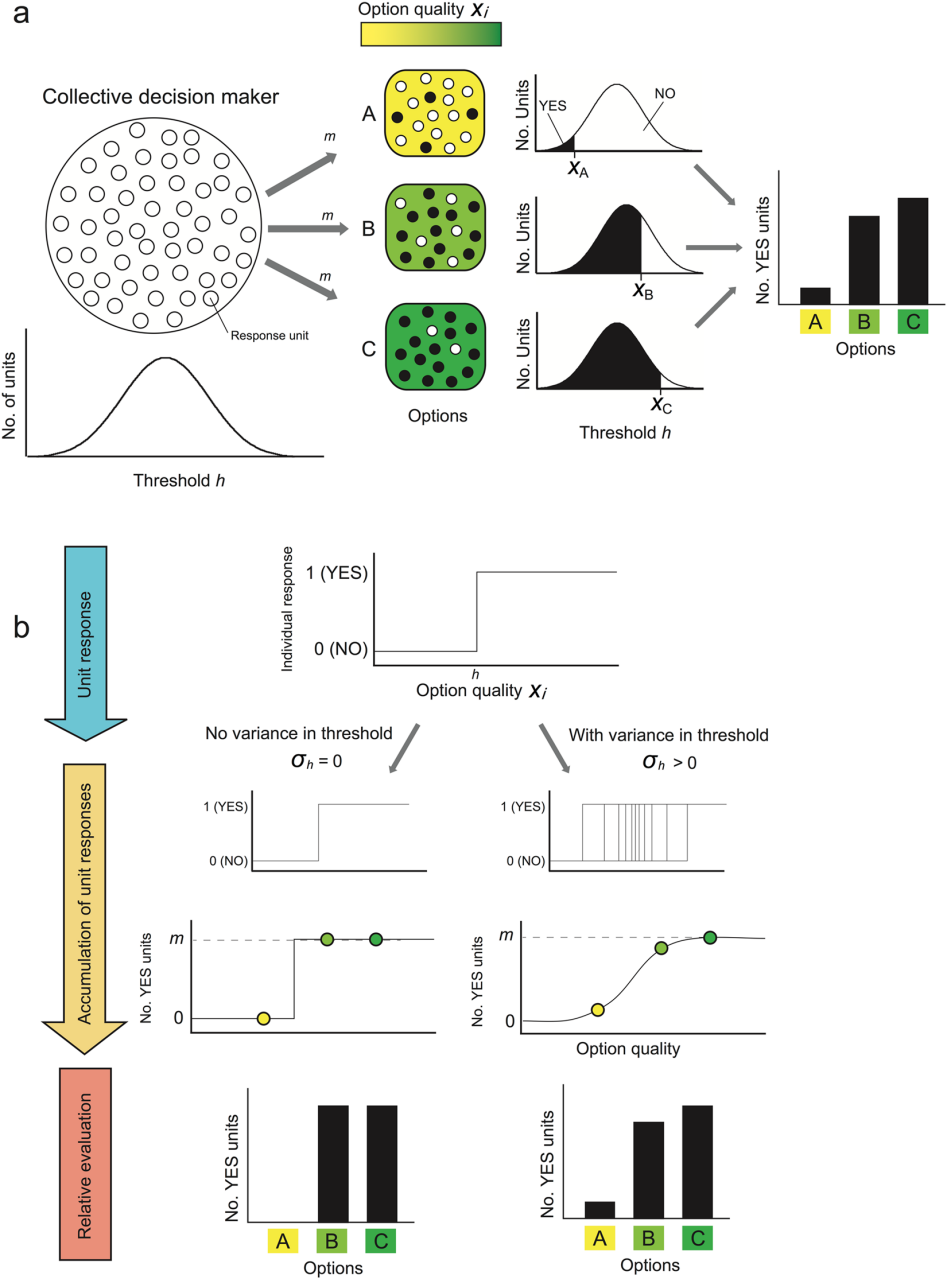
Discrimination mechanism for quality value of multiple options. (**a**) A scheme for discriminating the quality of three options. A collective decision maker allocated a number of units to each option. Each unit can make a “yes” response when the quality of the allocated option exceeds its threshold. Then, the collective decision maker chooses the option that acquired the largest number of “yes” responses by majority decision. If the chosen option had the highest quality, the collective decision maker succeeded in making a correct decision. (**b**) The need of variance in response thresholds to an option among allocated units to make the correct choice. If there is no variance among allocated units, the evaluation function has only a single step, and thus the collective decision maker cannot discriminate a difference in quality between two options that are the same step (left). When there is sufficient variance among units allocated to an option, the evaluation function becomes an increasing curve that enables a decision maker to discriminate differences in quality among options.

The notations used below are summarized in Table 1. We first explain the evaluation mechanism for relative quality among multiple options used by a collective decision maker that consists of simple yes/no judgement units^18^. Consider a situation in which a collective decision maker chooses the best option from multiple candidates. Set *n* options with different quality values (*x*_*i*_) and allocate *m* units to each option from the decision maker (Fig. 1a). A unit *j* assigned to an option *i* constitutes a “yes” response when the quality value (*x*_*i*_) of the option is higher than the threshold (*h*_*j*_) of the unit. Here, we did not consider any error in individual quality assessments by unit, and therefore, the evaluation function of unit *j* becomes a single step function (*F*_(*i,j*)_) (Fig. 1b). For these options, the decision maker counts the number of units that exhibit a “yes” response and chooses the option with the greatest number of “yes” responses (yes-majority decision). When all units of a decision maker have the same threshold value (no variance in response threshold values and no assessment error), they respond with “no” for options with a lower quality than *h* and respond with “yes” for options with a higher quality than *h* (Fig. 1c left). Thus, the collective evaluation function (*F*_(*i*)_) becomes a single step function (Fig. 1c left), where the decision maker cannot discriminate the values of two options if they are on the same side (Fig. 1d left). However, if the standard deviation (*σ*_*h*_) in threshold *h* is large enough, the collective evaluation function becomes a multi-step function (Fig. 1c right; see also Fig. 1 of ref.^21^), where the decision maker can easily distinguish the quality differences among options by counting the number of “yes” responses to each option. Thus, the decision maker can correctly estimate the quality order of the options if the standard deviation (*σ*_*h*_) and the number of units (*m*) are sufficiently large (Fig. 1d right). In this study, we defined a correct decision by the decision maker as a choice of the option with the highest quality from multiple candidates.

**Table 1:** List of notations used in this study.

Symbol	Definition	Default value(s)	Range
***n***	Number of options	2, 5, 10, 20	[2, 50]
***m***	Number of response units	100	[1, 100]
*D*_*m*_ *	Distribution of the number of response units allocated to each option; ~Normal(*m*, *σ*_*m*_)		
***σ***_***m***_	Standard deviation of *m*	0	[0, 30]
*h*_*j*_	Response threshold value of response unit *j*		
*D*_*h*_	Distribution of *h*; ~Normal(*μ*_*h*_, *σ*_*h*_)		
***μ***_***h***_	Mean of *h*	0	[–4, 4]
***σ***_***h***_	Standard deviation of *h*	1	[0, 4]
*x*_*i*_	Option quality value of option *i*		
*D*_*x*_	Distribution of *x*; ~Normal(*μ*_*x*_, *σ*_*x*_)		
***μ***_***x***_	Mean of *x*	0	[–4, 4]
***σ***_***x***_	Standard deviation of *x*	1	[0, 4]
***q***	Quorum		[0, 100]

**Figure 2:**
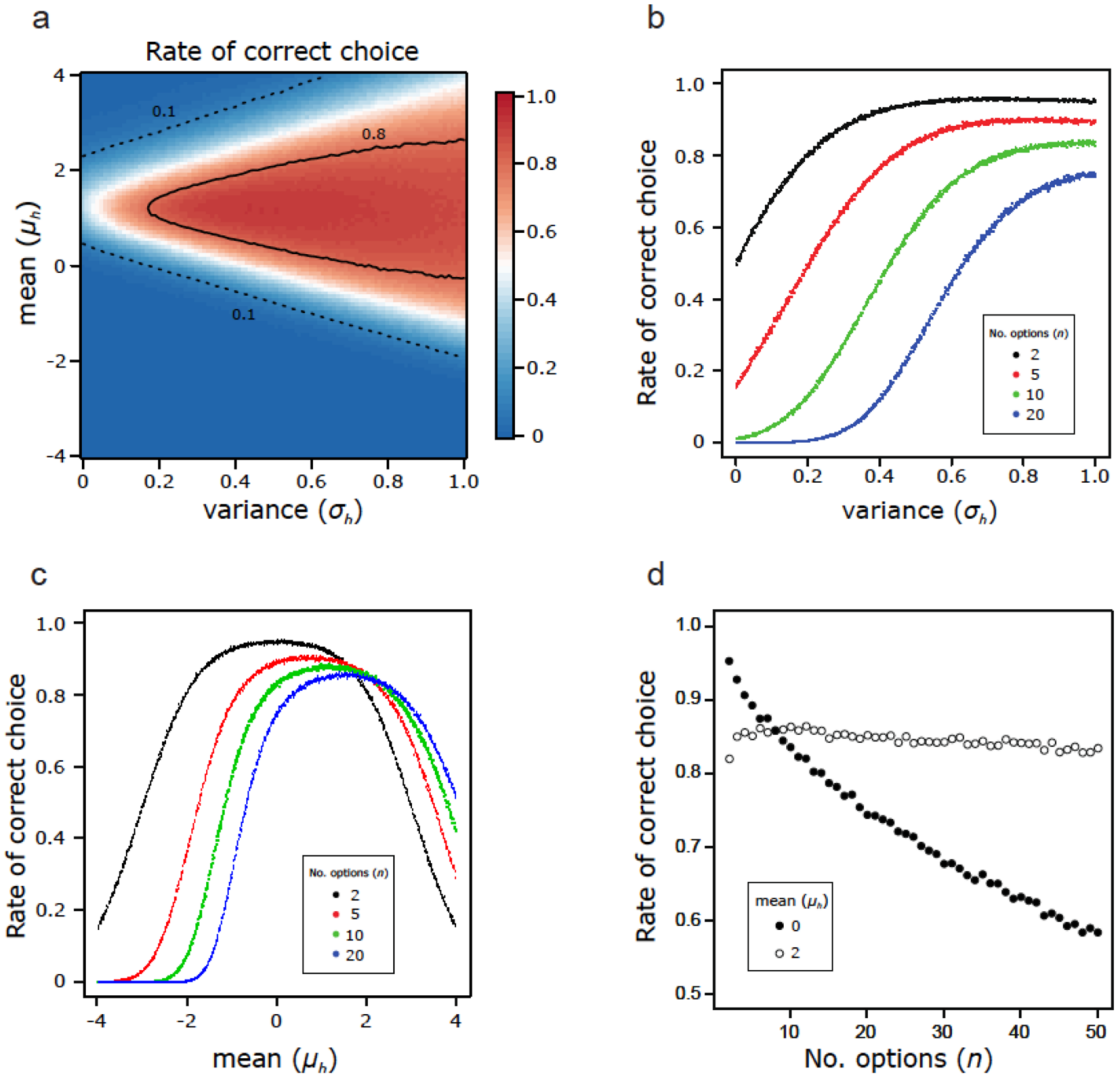
Best performing parameters of the response threshold distribution in yes/no unit groups. Response threshold values follow a normal distribution with mean *μ*_*h*_ and standard deviation *σ*_*h*_. Quality values of options follow a standard normal distribution. (**a**,**b**) The effect of different response threshold standard deviations on the proportion of correct choices. A high proportion of correct choices can be achieved with variance in the response threshold of units. (**a**,**c**) The effect of the mean response threshold on the proportion of correct choices. The results with 10 options are shown in (**a**). (**d**) The relationship between the number of options and the proportion of correct choice across different mean thresholds. Units that are more choosy (high mean) are needed to solve a difficult problem (i.e., relative evaluation of many options). The parameters that are not analysed here are set as default values in Table 1.

A simulation based on the above settings showed that a high proportion of correct choices can be achieved with sufficient variation in the unit threshold (Fig. 2a,b). When the standard deviation of the threshold was large, the proportion of correct choices was more than 80% (Fig. 2a,b). The proportion of correct choices was also affected by the mean (*μ*_*h*_) of the threshold value distribution of units within the decision maker (Fig. 2a,c). The proportion of correct choices first increased with the value of the mean and then decreased (i.e., unimodal). Our model also shows that more units with a high threshold (choosy units) are required to solve a difficult choice (i.e., choice from many options). When the threshold distribution has the same mean as the quality-value distribution of options (*μ*_*x*_ = *μ*_*h*_ = 0 in Fig. 2d), the proportion of correct choices decreases rapidly with the increase in the number of options (black circles in Fig. 2d). In an excessively choosy (*μ*_*x*_ = 0, *μ*_*h*_ = 2) case, as the number of options increases, the proportion of correct choices first increases and then decreases slightly (white circles in Fig. 2d). In summary, the proportion of correct choices is higher when the threshold distribution matches the quality-value distribution under a small number of options, but this proportion is reversed when the number of options becomes large (Fig. 2d). In other words, a difficult problem (many options) requires more choosy-units to solve. The high mean of the threshold distribution means that the thresholds of many units exist within a range where high-quality options are concentrated due to a large number of options. Thus, the evaluation function increases abruptly in that range, resulting in high resolution of the collective decision maker in that range.

**Figure 3:**
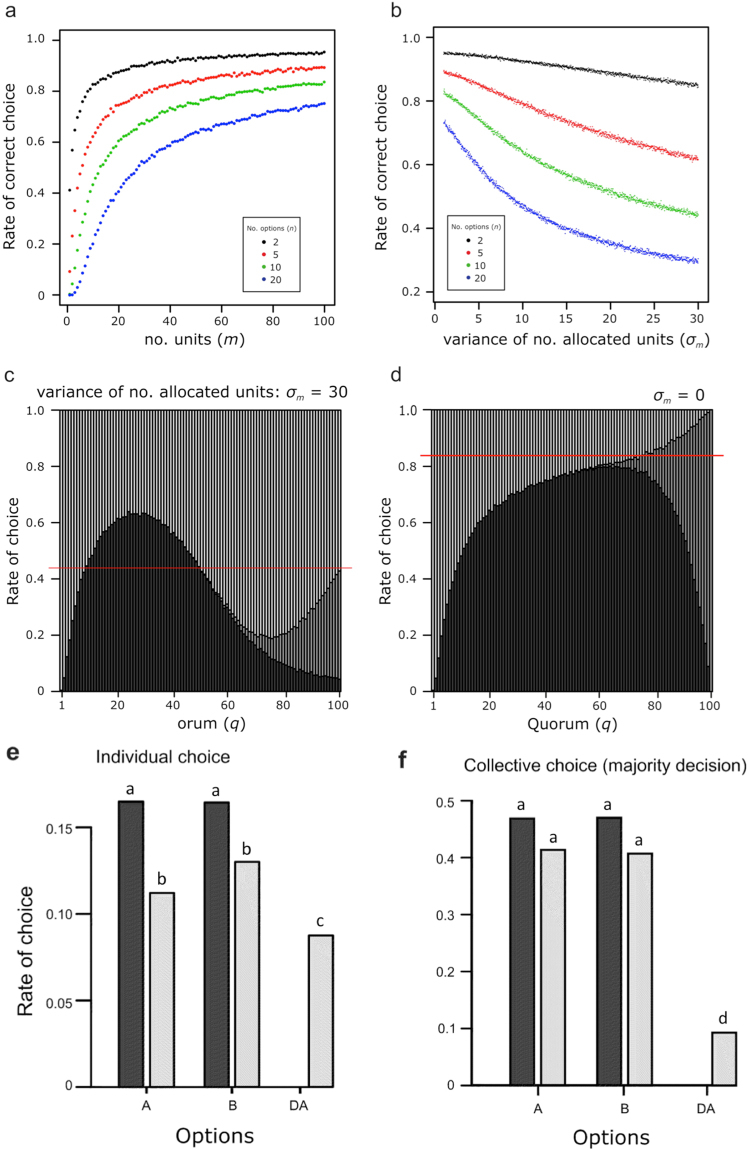
Simulation results showing how collective decisions are affected by differences in the number of units allocated to options. (**a**) A high proportion of correct choices with a large number of units. (**b**) Reduction in the number of correct choices according to increased standard deviation of the number of units allocated to each option (*σ*_*m*_). (**c**,**d**) Quorum decision improving the correct choice performance under such variation in the number of units among options. With variance in the number of units (*σ*_*m*_ = 30), decision-making by quorum decision exceeds that by majority decision, whereas with no variance (*σ*_*m*_ = 0), quorum decision is always inferior to the majority decision. The results with 10 options are shown. Black, grey, and white regions indicate correct choices, quorum not reached, and incorrect choices, respectively. Red lines indicate the proportion of correct choices by majority decision. (**e**,**f**) Outcome of binary choices between options A and B (black bars) and ternary choices among options A, B and D_A_ (grey bars) with the collective (majority) decision (**e**) and individual decisions (**f**). The parameters that are not analysed here are set as default values in Table S1.

The proportion of correct choices increases with the number of allocated units in each option (Fig. 3a) because the evaluation function becomes smooth (Fig. 1c right) under such a condition. In nature, the number of allocated units among options would vary. For example, it has been shown that the number of allocated honeybee scouts varies among available options^11^. In the case of *Temnothorax albipennis* ants, when colonies choose between a distant high-quality nest and a much closer but poorer quality next, more ants initially assess the closer nest^24^. Incorporating variation in the number of units into our model, the proportion of correct choices decreased with the degree of variation (Fig. 3b). This is because the decision maker estimates the quality of options based on the number of “yes” responses regardless of the number of “no” responses, which disturbs the correct relative evaluation of options. For example, 10 “yes” responses from 10 units allocated to the optimal option is underestimated relative to 20 “yes” responses from 100 units allocated to another option. To test whether this inevitable trap can be avoided, we conducted another simulation adopting quorum decision as observed in social insect colonies^11,24,25^. In this additional simulation, a quorum size (*Q*) was assigned equally to all the options, and the option that first exceeded the quorum was selected as the best option. We examined the proportion of correct choices along with quorum size. When the number of allocated units was varied among options, there was a region in which the performance of the quorum decision became superior to that of the yes-majority decision (Fig. 3c). Conversely, when there was no variance in the number of allocated units among options, the performance of the yes-majority decision always became superior to that of the quorum decision (Fig. 3d).

Next, we assessed whether our proposed collective decision-making mechanism by yes/no judgement units possesses the property of independence of irrelevant alternatives (IIA(L))^26,27^. IIA means that a preference of a decision maker for an option cannot change when an alternative option is added. If the decision maker is able to evaluate the option accurately, IIA would not be violated by an alternative option. It has been demonstrated that the preferences of groups of *Temnothorax* ants are not susceptible to the third option, but each individual ant is susceptible^9,28^. To examine this property, we modified the simulation as follows: options have two independent attributes with quality values, and units also have two independent response thresholds corresponding to these two types of option quality. We simulated the situation where a collective decision maker chooses one option from two options (A and B) that differed in two attributes, such that the preference of A and B must be equal for collective decision makers. Here we added the third inferior option (DA) that is asymmetrically dominated for the other two attributes (decoy), and tested whether the third option can affect the results of binary choice. This modified simulation showed that the existence of the third inferior option had no effect on the proportion of choosing the two options (A and B) in both collective decisions, with the majority decision (A:B = 4135:4069 with decoy and A:B = 4686:4702 without decoy in 10,000 trial; Fisher’s exact probability test: p = 0.5226; Fig. 3f) and quorum decision (p = 0.4424; data not shown). Then, to reveal the effect of another inferior option on the response of each unit, we modified the model as follows: each unit assesses all the options and picks one that satisfies its expectation (i.e., its quality is higher than its threshold). If there is more than one option that satisfies its expectation, then a unit chooses one of these randomly, which corresponds to the individual ant choice rule^9^. Simulating this individual choice of units with and without the decoy, the result was different from that of collective decision, where the existence of a decoy had significant effect on the proportion of choosing the two options (A:B = 11179:12974 with decoy and A:B = 16465:16408 without decoy in 100,000 trial; Fisher’s exact probability test: p < 0.0001; Fig. 3e). Thus IIA is violated in the individual decision but not in the collective decision for the current yes/no judgement decision mechanism. However the simulated violation was different from that observed in *Temnothorax* ants, in which individual ants exhibit decoy effects, that is, prefer A to B in the individual decision^9^.

**Figure 4:**
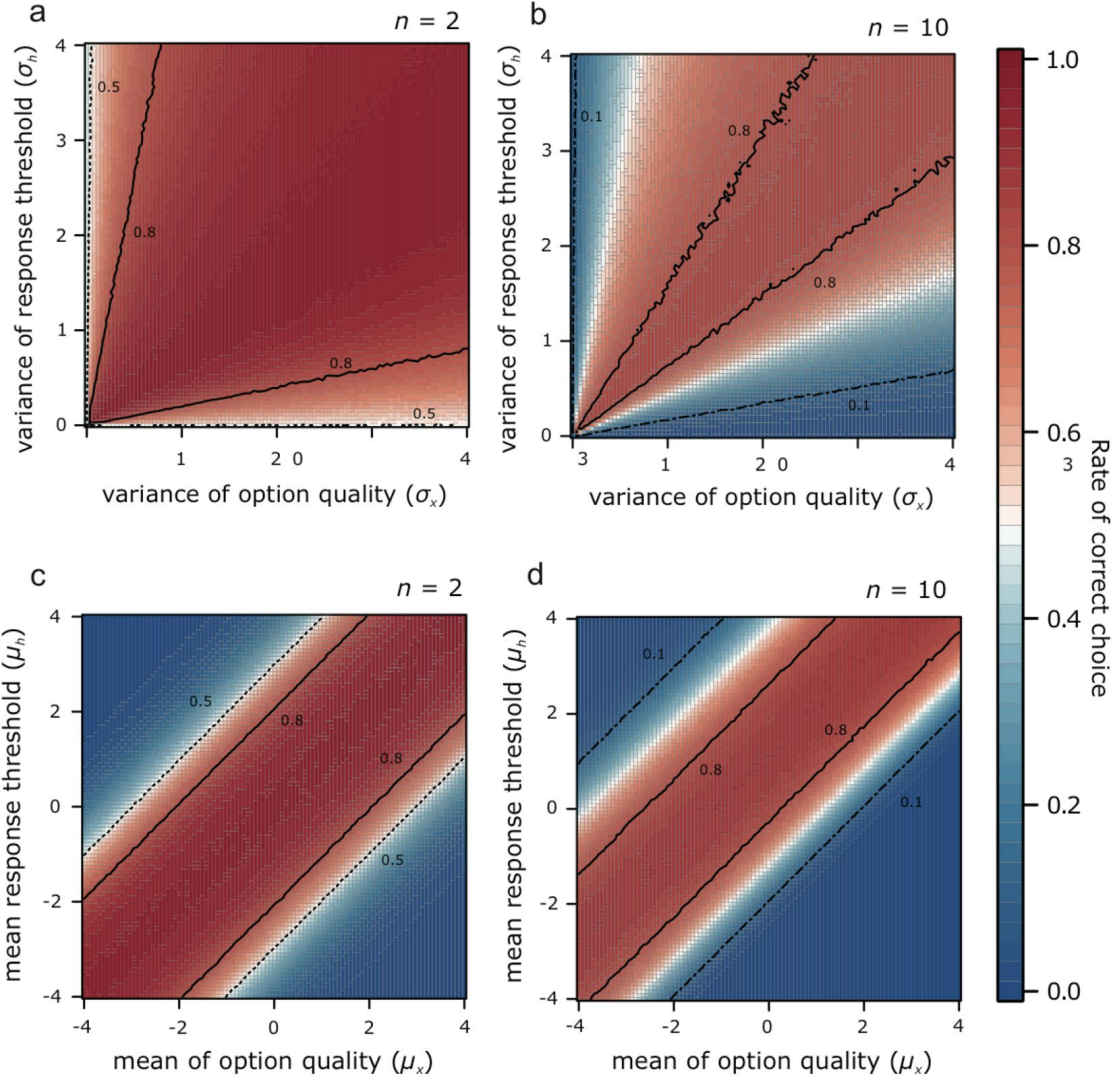
Simulation results implying the existence of an optimal response threshold distribution for the distribution of quality values of options. (**a**,**b**) The relationship between standard deviation of response threshold and the proportion of correct choices across the standard deviation of option quality. (**c**,**d**) The relationship between the mean response threshold and the proportion of correct choices across the mean option quality. The parameters that are not analysed here are set as default values in Table 1.

In all the previous simulations, the distribution of quality values of options was fixed to the standardized normal distribution (see Methods). Modification of the distribution of quality values showed that the distribution of threshold values that maximizes the proportion of correct choices was changed (Fig. 4). In the choice between two options, the proportion of correct choices was maximized when the distribution of the response threshold was similar to that of option quality (Fig. 4a,c). If the number of options was increased to 10, the quality value of the best option tended to increase. Thus, the mean of the threshold values that achieves high performance deviated towards the expected value of the best option (Fig. 4d), and the variation of threshold values that achieves high performance became slightly larger than that of the quality values (Fig. 4b). Note that we generally did not suppose any error in quality assessments, such as sensory noise, which is incorporated into the previous models focusing on collective decision-making by social insects^15,17^. When we perform simulations with assessment error, the proportion of the correct choice is hardly affected by the error, especially in the region where the error is smaller than the standard deviation of thresholds (Supplementary Fig. S1a).

## Discussion

Our results demonstrate that variation in the threshold values of allocated units is important for making a relative evaluation among options and, thus, the correct choice in collective decision-making. Even without a variation in response thresholds, a collective decision maker is able to choose the optimal option when the best option alone is always the right side of the step (i.e., the quality value of the best option alone is higher than the threshold of the units). This is possible when the distribution of option quality is predictable or constant. However, collective decision makers in the real world would often face multiple options with variable and unpredictable quality values. To solve this problem with high probability, our results show that high variation in threshold is important (Fig. 2a,b); it is especially important that the threshold distribution has the same form as the distribution of the option quality. This is because a variable threshold provides the collective decision maker with a smooth evaluation-function, which is essential for choosing the best option from multiple options with unpredictable and variable quality values.

Another problem of invariant thresholds is the deadlock problem in which more than one option has acquired the same largest number of “yes” responses, disturbing the collective decision. This problem can be solved by a random choice between these options with the same largest “yes” number (see Supplementary Fig. S1b) or by a signalling-stop as observed in honeybee swarms^29,30^. Most likely, because real organisms have no way to randomly select between options with the same best “yes” number, they use a signalling-stop to solve the deadlock problem. In any case, our simulations showed that a group of simple yes/no units can achieve the correct choice at high probability even when faced with multiple options with variable and unpredictable quality values.

The concept of threshold variance is derived from empirical studies of social insects^31–33^. Several recent studies have demonstrated that a simple yes/no response is sufficient for ant colonies to achieve highly efficient correct choices^20–22^. For example, in the case of nest-site selection by ant species, individual ants assess the visited option and recruit others only when the quality of the option exceeds their threshold^20^. Other model studies using simple yes/no units have well explained the speed-accuracy and speed-cohesion trade-offs observed in real ant colonies^22,23^, one of which assumed two groups with a low or a high threshold in a colony that made the correct choice^21^. A recent study revealed that when faced with two options with different qualities, the majority of units with thresholds between the two quality values choose the better option^21^. Ref.^22^ defined a distribution of thresholds to realize the above condition, and their model succeeded. Ref.^22^ also showed that the proportion of correct choices increased along with the proportion of individuals with a high threshold (choosy individuals). However, our results show that excess choosy units decrease the proportion of correct choices (Fig. 2c). This difference would arise from the difference between the forms (binary versus normal) of the threshold distribution in the two studies. In fact, our study showed that the performance of the current mechanism is maximized by similarity between the form of the threshold distribution, especially in the choice between two options (Fig. 4a–c). In this context, our model predicts that ants with adequate variations in response thresholds can efficiently recruit others to the high quality option even from among multiple options with unpredictable and variable quality values. Moreover, we revealed the features of collective choice by yes/no response units, including the existence of an optimal threshold distribution for units depending on the number of options and the distribution of quality values. The highest performance must be achieved when the threshold values of allocated units are distributed between the quality values of the best and second- best options, as a previous study showed that the majority of individuals with thresholds between two options makes the correct choice^21^. Integrating these results, our model predicts that the distribution of response thresholds in social insects is tuned to the distribution of resource quality in their environment.

One problem in the current mechanism for correct decision-making is the effect of assessment errors by units. Real animals may make a wrong assessment regarding an option’s quality, but our simulation revealed that such assessment errors only minimally affected the correctness of the collective choice (Fig. S1a). However, an initial wrong assessment by the current mechanism is possible. Thus, for ants and honeybees, colonies use “quality-graded responses” in addition to response threshold variance in their collective decisions to assure the correct choice^5,11,12,16^. The observed combination may be an assurance system by which a colony can cope with a speedy choice of an option by the current mechanism and correction of a possible wrong choice using only the current mechanism. Further studies are required to examine the relationship between the two mechanisms.

Our results are in accordance with the observation in ant colonies in which individual decision-making is superior to collective decision-making when a task is easy but the opposite is true when a task is difficult^34^. Here, “easy” indicates that the differences between options in terms of quality are large, and “difficult” means they are small. A large number of options inevitably results in small option-quality differences, i.e., “difficult”. A larger number of units would make it easy to discriminate small differences in quality among options because it increases the probability of the existence of units that can discriminate the quality difference between any pair of options. Thus, more scouting units would result in a more precise decision than the decision made by a small number of units.

The results of the modified simulations with two different attributes showed that the preference in binary choice is not affected by the existence of a third inferior option in collective decisions (see Results section and Fig. 3e). This suggests that the current mechanism of collective decision by simple yes/no units possesses the property of IIA, which confirms that our mechanism enables a collective decision maker to evaluate the quality values of multiple options without direct comparisons. Interestingly, in our simulation focusing on the choices of individual units, the third inferior option affected the choice made by some units (Fig. 3f), but it was in the opposite direction as reported in the previous results of *Temnothorax* ants^9^. When an individual chooses from the options A, B and D_A_, the presence of option D_A_ makes option A less popular (Fig. 3f) whereas option A is more popular in ants^9^. In our model, some units choosing option A under the condition without D_A_ changed their choice to D_A_ because A and D_A_ have indistinguishable quality values for the units. Thus a single yes/no unit can violate IIA and be considered irrational when it makes a decision. Based on these results, we have demonstrated that rational decision-making can emerge from the collection of irrational individuals using our mechanism.

The optimality of collective decision-making has been discussed from a different context in previous studies than in this study. For example, previous studies^15,22^ have considered how a collective decision maker can manage the speed-accuracy trade-off when it chooses one from among two options. Another study analysed the required conditions for a majority decision to work well for a binary choice by individuals who choose the correct choice with a given probability^13^. A further study examined the performance of collective decision-making in the context of a trade-off between achieving a high true positive rate and a low false positive rate, for which setting quorums appropriately is an important factor^14^. In these studies, the proportion of correct choices based on collective decision is dependent on the probability of finding the correct answer by a judgement unit. When each unit has greater than 50% probability of making the correct judgement, the majority decision becomes more precise with the number of assessed units, which is known as “Condorcet’s jury theorem“^35^. However, this theorem requires the probability of correct judgement to be greater than 50% for each unit. If each unit’s probability is less than 50%, a majority decision tends to choose the wrong option. Our results showed that it is most important how a judgement unit can achieve a probability of correctly evaluating an option of greater than 50%. The answer is using a group of yes/no units with variable response thresholds as judgement units, similar to the mechanism of the brain^36^.

In contrast to these previous studies, this study focused on accurately evaluating the relative quality of multiple options by simple yes/no units (without more than 50% probability of a correct judgement) and provided simulation models to reveal that this relative evaluation system enables collective decision-makers to choose the highest quality option via a majority decision-making process. We also extensively analysed the conditions under which a collective decision maker with simple yes/no units can efficiently achieve a correct choice. Thus, our study added a novel mechanism for how sophisticated decision-making can emerge from a group of simple yes/no units into the field of collective decision-making. A previous study on collective decision-making in ants revealed that groups have a larger cognitive capacity than individuals^37^. All animals are believed to have lower cognitive abilities than human beings. Therefore, grouping animals and using collective decision-making may be a better way to make decisions for the group as a whole than to make individual decisions. The current mechanism provides new insight into the emergence of rational collective decision-making.

Finally, our system can be applied to various fields. The brain is a system that includes groups of neurons that make simple on/off responses to a stimulus to achieve adaptive judgements in response to changing environmental conditions^15^. This study contributes to elucidating the mechanism of the model used by the brain. Several studies have shown consistency in the assumption that monkeys’ brains use the current system to code the value of resources^36,38^. In brain regions that assess the quality of options, more neurons fire as the quality of an option increases, and the rate of firing in response to an option’s quality is not influenced by the existence of other options^15,36,38^. These findings suggest that a brain assesses the value of a resource by using a group of neurons for each option. Thus, the current mechanism examined in this study provides important insights into various phenomena in neuroscience. It can also be applied to group decision-making in operations research. Analytic hierarchy processes (AHPs) can solve the ranking problem in which the complete order of multiple options is estimated based in multiple people, each of which provides part of the information needed to order the options^39^. In our system, each person ( = unit) only provides a yes/no judgement for a single option. Therefore, without a person comparing among options, our system can generate the order of multiple options as long as enough people are engaged in the judgement task. In this study, as a person (i.e., a single unit) is engaged in the judgement of a single option, our system can be used as a new method for market monitoring in management science^40,41^. We can determine the best merchandise among multiple candidates by asking only the following question to all the testers: “would you buy this merchandise at this price?” The best merchandise is the product for which the largest number of people answer “yes”. Although we tend to believe that choosy units are better for making the correct choice, our results showed that the proportion of correct choices sometimes decreased in such cases (Fig. 2c). In conclusion, we showed that the relative evaluation of options can emerge from a group of yes/no units with variations in threshold values and that this system ensures that the correct choice is selected from among multiple candidates given a sufficient number of units and variation in threshold. Of course, real groups of animals use “quality-graded responses” (the degree of response increases with the quality of an option) to achieve collective rationality^5,10–12,15^. Thus, many realized collective systems in nature and human societies may be governed by the current proposed systems that integrate quality-graded responses.

## Materials and Methods

### Basic model using majority decision

We developed individual-based models to investigate the general features of collective decision-making by simple yes/no decision units with response-threshold variability. We considered a situation in which a group of simple yes/no units (a decision maker) chooses the best option from among multiple candidates. The selection process was as follows. Set *n* options with different quality values and allocate *m* units to each option from the decision maker. Each allocated unit *i* assesses option *j* and makes a “yes” response when the quality value of option *x*_*i*_ (from a normal distribution with a mean and standard deviation (*μ*_*x*_, *σ*_*x*_): abbreviated as [~Normal (*μ*_*x*_, *σ*_*x*_)]) is higher than the threshold of unit *h*_*j*_ ~ Normal (*μ*_*h*_, *σ*_*h*_). In general, we considered that there was no error in responses by units in this evaluation process, but we also analysed the situation with evaluation errors, which would be a more accurate approximation of the real world. For this simulation, we added error *ε* to each quality of option ~Normal (*μ*_ε_ = 0, *σ*_ε_ = 0.1). After all units respond to their options, the decision maker counts the number of units that show a “yes” response for all options. Then, the decision maker chooses the option that received the largest number of “yes” responses. Thus, the decision maker adopts the majority decision. When the chosen option had the highest quality among options, we regarded that the collective decision maker succeeded in the correct choice. If more than one option had received the largest number of “yes” responses, we considered that the decision maker failed to make the correct choice because under this condition, the decision maker could not select an option by majority decision. For simulations in which the number of allocated units varied among options, the number of allocated units was varied by rounding up the values ~Normal (*N*_*m*_, *σ*_*m*_). If the value was less than 0, we treated it as 0.

### Modified model using quorum decision

In addition to the simulations with majority decision, we conducted another simulation based on a quorum decision. In this simulation, a quorum *q* was given to the collective decision maker, and the option that first exceeded the quorum was selected as the best option. To model this decision process, we assumed that each allocated unit assesses options one by one. When the number of “yes” responses to an option exceeded the quorum, we recorded the number of units that responded “yes” to that option, and compared the number of “yes” responses for each option that had acquired more “yes” responses than the quorum. The decision maker then chooses the option that required the smallest number of units to exceed the quorum. If no option exceeded the quorum or if there was more than one option that required the smallest number of units, we considered that the decision maker failed to choose the best option.

### Violation of Independence of Irrelevant Alternatives

We also examined the effect of another inferior option (decoy) on our model. To simulate a decoy, we assumed that options have two independent attributes, each with a different quality value. Similarly, units also have two independent response thresholds corresponding to these two option qualities. Each response unit *i* assesses option *j* with two different attributes and makes a “yes” response when both of the quality values (*x*_*j*_ and *y*_*j*_) are higher than the corresponding thresholds of the unit (*h*_*x, i*_ and *h*_*y, i*_). For binary choices (without decoy), the decision maker chooses one of two options, A or B, designed such that neither attribute is completely superior to the other (e.g., A: *x*_*A*_ = 0.2 and *y*_*A*_ = 0; B: *x*_*B*_ = 0 and *y*_*B*_ = 0.2). For ternary choices (with a decoy), the decision maker chooses one of three options, A, B or a decoy (D_A_). The decoy D_A_ is dominated by A but not by B (D_A_: *x*_*DA*_ = 0.2 and *y*_*DA*_ = −0.2). We tested whether presence of the third option (D_A_) can affect the preference of two options (A and B) with two decision mechanisms: a majority decision and a quorum decision where we set the value of quorum *q* to 60.

Next, we focused on the effect of a decoy on the response of individual units. To reveal the features of individual units, we performed a small modification of the collective decision model simulation with a decoy—each unit assesses all the options and picks one that satisfies its expectation (i.e., both qualities are higher than their thresholds). If there is more than one option that satisfies its expectation, then a unit chooses one randomly among them. In this simulation, we assessed individual responses of the units, which is the condition representing when individual ants make a decision alone (p. 279 1st paragraph of ref.^9^).

### Simulations

For each parameter set, we ran 10000 simulations and recorded which options the decision maker chose. Default values and ranges of parameters are listed in Table S1. We performed all simulations using R version. 3.1.2 (R: a language and environment for statistical computing; Development Core Team, Vienna, Austria).

### Data and materials availability

The source codes of all the simulations reported in this paper are described in the Supplementary Materials.

## Electronic supplementary material

Supplementary information
