# Supplementary material for: Nature of collective decision-making by simple yes/no decision units
Source: Sci Rep. 2017 Oct 31;7:14436. doi: 10.1038/s41598-017-14626-z (PMC5663756; doi:10.1038/s41598-017-14626-z)
Supplement: Supplementary file 1 — Supplementary information [file 41598_2017_14626_MOESM1_ESM.doc]

**Supporting Information file**

**Title:** Nature of rational collective decision-makings by simple yes/no decision units.

**Authors:** Eisuke Hasegawa^1^*, Nobuaki Mizumoto^2^, Kazuya Kobayashi^2†^, Shigeto Dobata^2^, Jin Yoshimura^3,4,5^, Saori Watanabe^1^, Yuuka Murakami^6^, Kenji Matsuura^2^

^1^ Laboratory of Animal Ecology, Department of Ecology and Systematics, Graduate School of Agriculture, Hokkaido University. Sapporo 060-8589, Japan.

^2^ Laboratory of Insect Ecology, Graduate School of Agriculture, Kyoto University, Kyoto 606-8502, Japan.

^3^ Graduate School of Science and Technology and Department of Mathematical and Systems Engineering, Shizuoka University, 3-5-1 Johoku, Naka-ku, Hamamatsu 432-8561, Japan.

^4^ Marine Biosystems Research Center, Chiba University, Uchiura, Kamogawa, Chiba 299-5502, Japan.

^5^ Department of Environmental and Forest Biology, State University of New York College of Environmental Science and Forestry, Syracuse, NY13210 USA.

^6^ Graduate School of Medicine, Department of Neuropharmacology, Hokkaido University. Sapporo 060-8638, Japan.

^†^ Present Address: Hokkaido Forest Research Station, Field Science Education and Research Center, Kyoto University. 553 Tawa, Shibecha-cho, Kawakami-gun, Hokkaido 088-2339, Japan.

*Correspondence author:

Eisuke Hasegawa

Laboratory of Animal Ecology, Department of Ecology and Systematics, Graduate School of Agriculture, Hokkaido University. Sapporo 060-8589, Japan.

e-mail:ehase@res.agr.hokudai.ac.jp

Tel: +81-11-706-3690

Fax:+81-11-706-2495


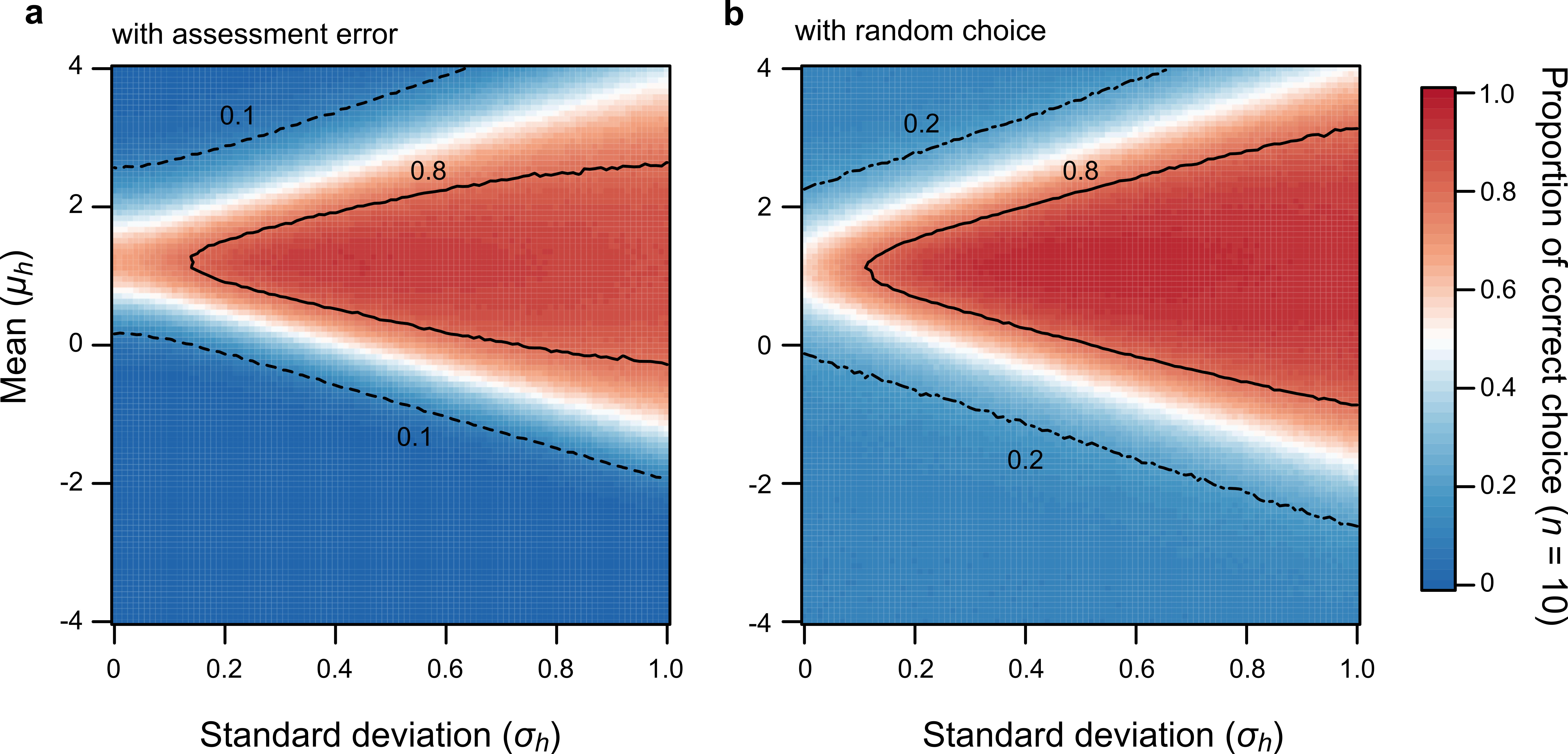


**Figure S1**

Simulation results with additional factors for collective choice among multiple options (*n* = 10). The simulated condition is the same with Figure 2**a**. (**a**) The effect of assessment error on the proportion of correct choice. The assessment error *ε* was incorporated by adding small noise sampled from normal distribution (*μ_ε_* = 0, *σ_ε_* = 0.1). (**b**) The effect of additional rule implemented to solve the problem of deadlock, that is, a collective decision maker cannot choose one since more than one options acquired the same largest number of "yes." Here we considered a random choice from these options with the same largest number of "yes.” Note that the overall proportion of correct choice was improved.

**# Simulation codes:**

# Nature of collective decision-making by simple yes/no decision units.

# Eisuke Hasegawa*, Nobuaki Mizumoto, Kazuya Kobayashi, Shigeto Dobata, Jin Yoshimura, Saori Watanabe, Yuuka Murakami, Kenji Matsuura.

# * Corresponding authors: Eisuke Hasegawa (ehase@res.agr.hokudai.ac.jp)

# Source code for R (version. 3.1.2)

### notations

# obj.value : values of options

# Thresholds : thresholds of units

# n.units : the number of units allocated to a single option (n)

# n.option : the number of options (m)

# myu.units = μh : mean of the distribution of thresholds

# sigma.units = σh : standard deviation of the distribution of thresholds

# myu.option = μx : mean of the distribution of values

# sigma.option = σx : standard deviation of the distribution of values

# param : number of partition in the range of focused parameters

# repeat.num : the number of replication for a single parameter set

# sig.m = σm : standard deviation of the numbers of units allocated to a single option

# answer : count the number of units that show "yes" response for each the option

# Quorum = quorum(Q) :

### Basic model using Majority rule

multchoice <- function(n.units, n.option, myu.units, sigma.units, myu.option, sigma.option){

obj.value <- rnorm(n.option, myu.option, sigma.option) # set the quality values of options from normal distribution (n, mean, sigma)

obj.value <- obj.value[order(obj.value)] # reordered

Thresholds <- matrix(rnorm(n.units * n.option, myu.units, sigma.units), ncol=n.units) # set the response thresholds of units

answer <- apply((Thresholds < obj.value),1,sum) # count the number of units that show "yes" response for each the option

return(as.numeric(sum(answer[1:(n.option-1)] < answer[n.option]) == (n.option -1))) # judge whether the decision maker chooses the optimal option (success: 1, fail: 0)

#we define the success as the optimal option get the largest number of "yes" response (see model section)

}

### Model using Majority rule with assessment error

multchoice_with_error <- function(n.units, n.option, myu.units, sigma.units, myu.option, sigma.option){

obj.value <- rnorm(n.option, myu.option, sigma.option) # set the quality values of options from normal distribution (n, mean, sigma)

obj.value <- obj.value[order(obj.value)] # reordered

Thresholds <- matrix(rnorm(n.units * n.option, myu.units, sigma.units), ncol=n.units) # set the response thresholds of units

Error <- matrix(rnorm(n.units * n.option, 0, 0.1), ncol=n.units) # set the response thresholds of units

answer <- apply((Thresholds < obj.value+Error),1,sum) # count the number of units that show "yes" response for each the option

return(as.numeric(sum(answer[1:(n.option-1)] < answer[n.option]) == (n.option -1))) # judge whether the decision maker chooses the optimal option (success: 1, fail: 0)

#we define the success as the optimal option get the largest number of "yes" response (see model section)

}

### Model using Majority rule with random choice

multchoice_with_random <- function(n.units, n.option, myu.units, sigma.units, myu.option, sigma.option){

obj.value <- rnorm(n.option, myu.option, sigma.option) # set the quality values of options from normal distribution (n, mean, sigma)

obj.value <- obj.value[order(obj.value)] # reordered

Thresholds <- matrix(rnorm(n.units * n.option, myu.units, sigma.units), ncol=n.units) # set the response thresholds of units

answer <- apply((Thresholds < obj.value),1,sum) # count the number of units that show "yes" response for each the option

return(as.numeric(sample(c(1,rep(0,sum(answer[n.option] <= answer[1:(n.option-1)]))),1))) # judge whether the decision maker chooses the optimal option (success: 1, fail: 0)

#if more than one option gained the largest number of yes, one is choisen randomly.

}

## example.1: investigate the effect of threshold distribution (μh and σh) on the rate of correct choice

param <- 101

repeat.num <- 10000

n.units <- 100

n.option <- 10

myurange<- seq(-4,4,length.out=param) # μh = 0~4

sigmarange <- seq(0,1,length.out=param) # σh = 0~1

res <- matrix(0, ncol = param, nrow = param) # storage of the results

for(k in 1:param){

for(j in 1:param){

for(i in 1:repeat.num){

res[j,k] <- multchoice(n.units, n.option, myurange[k], sigmarange[j], 0, 1) + res[j,k]

}

}

}

## example.1.1: investigate the effect of threshold distribution (μh and σh) on the rate of correct choice (with assessment error)

param <- 101

repeat.num <- 10000

n.units <- 100

n.option <- 10

myurange<- seq(-4,4,length.out=param) # μh = 0~4

sigmarange <- seq(0,1,length.out=param) # σh = 0~1

res <- matrix(0, ncol = param, nrow = param) # storage of the results

for(k in 1:param){

for(j in 1:param){

for(i in 1:repeat.num){

res[j,k] <- multchoice_with_error(n.units, n.option, myurange[k], sigmarange[j], 0, 1) + res[j,k]

}

}

}

## example.1.2: investigate the effect of threshold distribution (μh and σh) on the rate of correct choice (with random choice)

param <- 101

repeat.num <- 10000

n.units <- 100

n.option <- 10

myurange<- seq(-4,4,length.out=param) # μh = 0~4

sigmarange <- seq(0,1,length.out=param) # σh = 0~1

res <- matrix(0, ncol = param, nrow = param) # storage of the results

for(k in 1:param){

for(j in 1:param){

for(i in 1:repeat.num){

res[j,k] <- multchoice_with_random(n.units, n.option, myurange[k], sigmarange[j], 0, 1) + res[j,k]

}

}

}

## example.2: the simulations with a variance in the number of units allocated to a single option

param <- 1001

repeat.num <- 10000

n.units <- 100

sig.m <- seq(1,30,length.out=param)

res <- matrix(0, ncol = 4, nrow = param)

for(k in 1:4){

n.option <- c(2,5,10,20)[k]

answer <- rep(0, n.option)

for(j in 1:param){

for(i in 1:repeat.num){

obj.value <- rnorm(n.option, 0, 1) # set the quality values of options from normal distribution (n, mean:0, sigma:1)

obj.value <- obj.value[order(obj.value)] # arranged in order of increasing

A <- ceiling(rnorm(n.option, n.units, sig.m[j])) # creat the number of units allocated each option by rounding up the random value

for(h in 1:n.option){

if(A[h]<0){

A[h]<-0;

}

Thresholds <- rnorm(A[h], 0, 1) # set response thresholds of the units allocated the option h

answer[h] <- sum(Thresholds < obj.value[h])

}

res[j,k] <- as.numeric(sum(answer[1:(n.option-1)] < answer[n.option]) == (n.option-1)) + res[j,k]

}

}

}

### quorum decision (modified from example.2)

param <- 100

repeat.num <- 10000

n.units <- 100

n.option <- 10 # fixed

sig.m <- c(0,30) # check two extreme conditions

Quorum <- seq(1,100,length.out=param)

res <- matrix(0, ncol = 2, nrow = param) # storage of the number of correct choice

resn <- matrix(0, ncol = 2, nrow = param) # storage of the number of wrong choice

for(k in 1:2){

for(j in 1:param){

for(i in 1:repeat.num){

required.num.units <- rep(0,n.option)

obj.value <- rnorm(n.option, 0, 1)

obj.value <- obj.value[order(obj.value)]

A <- ceiling(rnorm(n.option, n.units, sig.m[k]))

for(h in 1:n.option){

if(A[h]<0){

A[h]<-0;

}

Thresholds <- rnorm(A[h], 0, 1) # set response thresholds of the units allocated the option h

answer <- 0

while(answer < Quorum[j]){

if (required.num.units[h] == A[h]) break # don't excess the quorum for the option h by the allocated units

if(obj.value[h] > Thresholds[required.num.units[h]+1]){

answer <- answer+1

}

required.num.units[h] <- required.num.units[h]+1 # count the number of requared units to excess the quorum for the option h

}

}

if(sum(required.num.units[1:n.option] != A[1:n.option]) == 0){ # all options don't excess the quorum

resn[j,k] <- 1 + resn[j,k]

}else{

res[j,k] <- as.numeric(sum(required.num.units[1:(n.option-1)] > required.num.units[n.option]) == (n.option -1)) + res[j,k]

}

}

}

}

### decoy effect

repeat.num <- 10000

n.units <- 100

myu.units <- 0

sigma.units <- 1

threshold1 <- rep(0,n.units)

threshold2 <- rep(0,n.units)

obj.value1 <- c(0.2, 0, 0.2) ## A, B, DA

obj.value2 <- c(0, 0.2, -0.2) ## A, B, DA

res <- rep(0,repeat.num)

## Majority Decision

res.without.decoy <- rep(0,repeat.num)

res.with.decoy <- rep(0,repeat.num)

# without decoy

for(i in 1:repeat.num){

threshold1 <- matrix(rnorm(n.units*2, myu.units, sigma.units), ncol=2)

threshold2 <- matrix(rnorm(n.units*2, myu.units, sigma.units), ncol=2)

answer <- apply((threshold1 < rep(obj.value1[1:2],each=n.units) & threshold2 < rep(obj.value2[1:2],each=n.units)),2,sum)

if(sum(answer == max(answer)) > 1){next()}

res.without.decoy[i] <- seq(1,2,1)[answer == max(answer)]

}

# with decoy

for(i in 1:repeat.num){

threshold1 <- matrix(rnorm(n.units*3, myu.units, sigma.units), ncol=3)

threshold2 <- matrix(rnorm(n.units*3, myu.units, sigma.units), ncol=3)

answer <- apply((threshold1 < rep(obj.value1,each=n.units) & threshold2 < rep(obj.value2,each=n.units)),2,sum)

if(sum(answer == max(answer)) > 1){next()}

res.with.decoy[i] <- seq(1,3,1)[answer == max(answer)]

}

## Quorum Desicion

quorum <- n.units * 0.6

# without decoy

for(i in 1:repeat.num){

answer <- rep(0,2)

threshold1 <- matrix(rnorm(m*2, myu.units, sigma.units), ncol=2)

threshold2 <- matrix(rnorm(m*2, myu.units, sigma.units), ncol=2)

answers <- ((threshold1 < rep(obj.value1[1:2],each=m) & threshold2 < rep(obj.value2[1:2],each=m)))

for(j in 1:K){

answer <- answer + as.numeric(answers[j,])

if(max(answer)>quorum){break()}

}

if(sum(answer == max(answer)) > 1){next()}

res.without.decoy[i] <- seq(1,2,1)[answer == max(answer)]

}

# with decoy

for(i in 1:repeat.num){

answer <- rep(0,3)

threshold1 <- matrix(rnorm(n.units*3, myu.units, sigma.units), ncol=3)

threshold2 <- matrix(rnorm(n.units*3, myu.units, sigma.units), ncol=3)

answers <- ((threshold1 < rep(obj.value1,each=n.units) & threshold2 < rep(obj.value2,each=n.units)))

for(j in 1:K){

answer <- answer + as.numeric(answers[j,])

if(max(answer)>quorum){break()}

}

if(sum(answer == max(answer)) > 1){next()}

res.with.decoy[i] <- seq(1,3,1)[answer == max(answer)]

}

## individual with decoy

N = 100000 # number of iterations

thresholds_x <- rnorm(N,0,1)

thresholds_y <- rnorm(N,0,1)

accept_xA <- rep(NA, N)

accept_yA <- rep(NA, N)

accept_A <- rep(NA, N)

accept_xB <- rep(NA, N)

accept_yB <- rep(NA, N)

accept_B <- rep(NA, N)

accept_xDA <- rep(NA, N)

accept_yDA <- rep(NA, N)

accept_DA <- rep(NA, N)

choice_binary <- rep(NA, N)

choice_ternary <- rep(NA, N)

##binary choice (w/o decoy)

for (i in 1:N){

if (thresholds_x[i] < 0.2){

accept_xA[i] <- 1

}else{

accept_xA[i] <- 0

}

}

for (i in 1:N){

if (thresholds_y[i] < 0){

accept_yA[i] <- 1

}else{

accept_yA[i] <- 0

}

}

for (i in 1:N){

if (thresholds_x[i] < 0){

accept_xB[i] <- 1

}else{

accept_xB[i] <- 0

}

}

for (i in 1:N){

if (thresholds_y[i] < 0.2){

accept_yB[i] <- 1

}else{

accept_yB[i] <- 0

}

}

for (i in 1:N){

if (accept_xA[i] == 1 && accept_yA[i] == 1){

accept_A[i] <- 1

}else{

accept_A[i] <- 0

}

}

for (i in 1:N){

if (accept_xB[i] == 1 && accept_yB[i] == 1){

accept_B[i] <- 1

}else{

accept_B[i] <- 0

}

}

choice_listAB <- c("A", "B")

for (i in 1:N){

if (accept_A[i] == 1 && accept_B[i] == 1)

{choice_binary[i] <- sample(choice_listAB,1)}

else

if (accept_A[i] == 1 && accept_B[i] == 0)

{choice_binary[i] <- "A"}

else

if (accept_A[i] == 0 && accept_B[i] == 1)

{choice_binary[i] <- "B"}

else

{choice_binary[i] <- "No choice"}

}

##ternary choice (w/ decoy)

for (i in 1:N){

if (thresholds_x[i] < 0.2)

{accept_xDA[i] <- 1}

else

{accept_xDA[i] <- 0}

}

for (i in 1:N){

if (thresholds_y[i] < -0.2)

{accept_yDA[i] <- 1}

else

{accept_yDA[i] <- 0}

}

for (i in 1:N){

if (accept_xDA[i] == 1 && accept_yDA[i] == 1)

{accept_DA[i] <- 1}

else

{accept_DA[i] <- 0}

}

choice_listAB_DA <- c("A", "B", "DA")

choice_listA_DA <- c("A", "DA")

choice_listB_DA <- c("B", "DA")

for (i in 1:N){

if (accept_A[i] == 1 && accept_B[i] == 1 && accept_DA[i] == 1)

{choice_ternary[i] <- sample(choice_listAB_DA,1)}

if (accept_A[i] == 0 && accept_B[i] == 0 && accept_DA[i] == 0)

{choice_ternary[i] <- "No choice"}

if (accept_A[i] == 1 && accept_B[i] == 0 && accept_DA[i] == 0)

{choice_ternary[i] <- "A"}

if (accept_A[i] == 0 && accept_B[i] == 1 && accept_DA[i] == 0)

{choice_ternary[i] <- "B"}

if (accept_A[i] == 0 && accept_B[i] == 0 && accept_DA[i] == 1)

{choice_ternary[i] <- "DA/Error"}

if (accept_A[i] == 1 && accept_B[i] == 1 && accept_DA[i] == 0)

{choice_ternary[i] <- sample(choice_listAB,1)}

if (accept_A[i] == 1 && accept_B[i] == 0 && accept_DA[i] == 1)

{choice_ternary[i] <- sample(choice_listA_DA,1)}

if (accept_A[i] == 0 && accept_B[i] == 1 && accept_DA[i] == 1)

{choice_ternary[i] <- sample(choice_listB_DA,1)}

}

CombinedTable <- data.frame(thresholds_x, thresholds_y, accept_xA, accept_yA,

accept_A, accept_xB, accept_yB, accept_B, choice_binary, choice_ternary) # make a table which has all the outcomes.

Tbinary <- table(factor(choice_binary, levels = c("A","B"))) # ignore "No choice"cases; if you want to include it, simply add "No choice" in the levels

Tternary <- table(factor(choice_ternary, levels = c("A","B", "DA"))) # ignore "nochoice" cases

par(mfrow=c(1,2), pin=c(3,3))

barplot(Tbinary, main = "binary", ylim=c(0,18000))

barplot(Tternary, main = "ternary", ylim=c(0,18000))
